# Evaluation of Mucoadhesive Gels with Propolis (EPP-AF) in Preclinical Treatment of Candidiasis Vulvovaginal Infection

**DOI:** 10.1155/2013/641480

**Published:** 2013-08-07

**Authors:** Andresa Aparecida Berretta, Patrícia Alves de Castro, Amanda Henriques Cavalheiro, Vanessa Silveira Fortes, Vinícius Pedro Bom, Andresa Piacezzi Nascimento, Franciane Marquele-Oliveira, Vinícius Pedrazzi, Leandra Naira Zambelli Ramalho, Gustavo Henrique Goldman

**Affiliations:** ^1^Faculdade de Ciências Farmacêuticas de Ribeirão Preto, Universidade de São Paulo, 14040-903 Ribeirão Preto, SP, Brazil; ^2^Laboratório de Pesquisa, Desenvolvimento e Inovação, Apis Flora Indl. Coml. Ltda., 14020-670 Ribeirão Preto, SP, Brazil; ^3^Faculdade de Odontologia de Ribeirão Preto, Universidade de São Paulo, 14040-903 Ribeirão Preto, SP, Brazil; ^4^Faculdade de Medicina de Ribeirão Preto, Universidade de São Paulo, 14040-040 Ribeirão Preto, SP, Brazil; ^5^Laboratório Nacional de Ciência e Tecnologia do Bioetanol-CTBE, Caixa Postal 6170, 13083-970 Campinas, SP, Brazil

## Abstract

Vulvovaginal candidiasis is the second cause of vaginal infection in the USA. Clinical treatment of *C. albicans* infections is routinely performed with polyenes and azole derivatives. However, these drugs are responsible for undesirable side effects and toxicity. In addition, *C. albicans* azole and echinocandin resistance has been described. Propolis is a bee product traditionally used due to its antimicrobial, anti-inflammatory, and other properties. Therefore, the present work aimed to evaluate different propolis presentations in order to evaluate their *in vitro* and *in vivo* efficacy. The methodologies involved antifungal evaluation, chemical analysis, and the effects of the rheological and mucoadhesive properties of propolis based gels. The obtained results demonstrated the fungicide action of propolis extracts against all three morphotypes (yeast, pseudohyphae, and hyphae) studied. The highest level of fungal cytotoxicity was reached at 6–8 hours of propolis cell incubation. Among the based gel formulations developed, the rheological and mucoadhesive results suggest that propolis based carbopol (CP1%) and chitosan gels were the most pseudoplastic ones. CP1% was the most mucoadhesive preparation, and all of them presented low thixotropy. Results of *in vivo* efficacy demonstrated that propolis based gels present antifungal action similar to clotrimazole cream, suggesting that future clinical studies should be performed.

## 1. Introduction

Vulvovaginal candidiasis is the second cause of vaginal infection followed by bacterial vaginosis in the United States of America. The costs involved with treatment, diagnosis, and the loss of labor productivity are around $ 1 billion per year. About 13 million prescriptions for treating fungal infections were made in 1990, and these numbers were about double those from 1980 [1]. *Candida albicans* is a fungal pathogen that is found as a commensal in humans and is the most common cause of mucosa and invasive fungal infections in humans. *C. albicans* is a pleomorphic microorganism that lives in the reproductive and gastrointestinal tracts in approximately half of the human population [2]. The balance between normal microflora is essential for health since when this status is interrupted or immunological defenses are compromised, *C. albicans* can be pathogenic due a transition in morphological state from yeast to hyphae. As a result, *C. albicans* infections are recognized as a serious challenge of public health with high social-economic and medical relevance [3]. Clinical treatment of *C. albicans* infections is routinely performed with polyenes, azole derivatives, allylamines, thiocarbamates, fluoropyrimidines, and echinocandins. However, these drugs are responsible for undesirable side effects and toxicity. In addition, *C. albicans* azole and echinocandin resistance has already been described [4–7]. Thus, considering the limited number of antifungal drugs and the continuous increase of *C. albicans* infection incidence, it is important to work continuously in the development of new drugs to treat this recurrent pathology, especially new drugs with high effectiveness and low adverse effects and costs.

Propolis is a complex mixture produced by honey bees, *Apis mellifera*, from the plant exudates, consisting of resinous and balsamic materials. The chemical composition includes flavonoids, terpenoids, phenylpropanoids, and many other compounds [8]. It has been reported that the flavonoids and the phenolic compounds are the main components responsible for the antibacterial, antiviral, and antifungal activities attributed to propolis extracts [9]. Many different extraction processes have been reported for propolis extracts, (e.g., alcoholic and aqueous) and currently, the pharmaceutical technology is focused on improving this field. Many extracts that function in different pharmaceutical applications, such as matrix microparticles and dry extracts, have been reported [10]. Therefore, the characterization of chemical and antifungal properties of these new options of propolis extracts can be valuable to the pharmacists who work with new products.

Recent reports have suggested that the market for mucoadhesive drug delivery systems is expanding rapidly [11]. Various administration routes, such as ocular, nasal, vaginal, rectal, and others, make mucoadhesive drug delivery systems attractive and flexible in dosage form development. The advantages associated with the use of mucoadhesives in drug delivery systems include increased dosage form, residence time, improved drug bioavailability, reduced administration frequency, simplified administration of a dosage form, and termination of a therapy as well as the possibility of targeting particular body sites and tissues [11]. In the present work, we investigated different pharmaceutical forms of propolis extracts (alcoholic, aqueous, microparticles, and dry extract) in order to choose one to compose mucoadhesive gels to treat vaginal candidiasis. These semisolid pharmaceutical presentations were assessed both *in vitro* and *in vivo*, and their efficacy as antifungal formulations against vulvovaginal candidiasis was demonstrated.

## 2. Materials and Methods

### 2.1. Chemicals

Purified water (Milli-Q); methanol HPLC grade (J. T. Baker, L.9093-68); formic acid (Vetec, L.0804789); caffeic acid (Fluka, L. 43706045); *ρ*-coumaric acid (Fluka, L.3250759); *trans*-cinnamic acid (Fluka, L.21907066); isosakuranetin (ChromaDex); artepillin C (Wako, L. 016.19131); 3,4-dicaffeoylquinic acid (3,4-DCQ) (Phytolab, L.13672938); 3,5-dicaffeoylquinic acid (3,5-DCQ) (Phytolab, L. 13672946); 4,5-dicaffeoylquinic acid (4,5-DCQ) (Phytolab, L. 13672903), *trans-*nerolidol (Sigma-Aldrich) gallic acid (Synth, L.109250); sodium bicarbonate (Vetec, L.0906112); and finally, aromadendrin-4′O-metil ether (previously isolated, identified and donated by Sousa et al. [12]) were employed.

### 2.2. Propolis Extracts

Propolis ethanolic extract (PEE), propolis water extract (PWE), propolis matricial microparticles (PMM), and propolis soluble dry extract (PSDE), were supplied by Apis Flora Indl. Coml. Ltda. (Ribeirão Preto, SP, Brazil). The extracts were obtained from a standardized propolis raw material (patent number PI0405483-0, Revista de Propriedade Industrial, n 1778, 2005). Since propolis is variable considering the plant vegetation of the area visited by honey bees, propolis standardized raw material was prepared in order to guarantee chemical reproducible composition of the propolis in study. For this, the company employed a mixture of propolis from different areas of Brazil, for example, Minas Gerais, São Paulo, Paraná, and Santa Catarina states, after the quality control of each one, considering several microbiological and physicochemical parameters and mainly the HPLC analysis, according to what was previously published by Berretta et al. [13]. Propolis blend of standardized raw material was previously cooled for 12 hours and pulverized. To obtain PEE, propolis raw material was macerated and percolated with hydroalcoholic solution (7 : 3) and finally filtrated.

PEE showed 11% w/v of extractable matter. PWE, PMM, and PSDE were obtained from the same PEE batch.

PMM was obtained by spray dryer employing PEE and excipients, such as modified corn starch (Capsul®) and colloidal silicon dioxide (1 : 1). The dryness condition involved temperature of 80°C for sample exit, sample flow of 12.0 mL/minute, and system air output of 3.0 m^3^/min. The spray dryer equipment used possessed a pneumatic atomizer with 1.2 mm of diameter. The ratio of dry matter and each excipient was 1 : 0.5 : 0.5, resulting in approximately 50% of propolis dry matter in PMM.

PSDE was produced after the dryness of PWE obtained according to de Andrade et al. [14] with modifications. PEE was submitted to complete evaporation of the solvent and to alkaline hydrolysis, followed by purified water addition (PWE). Subsequently, PWE was submitted to dryness with the presence of maltodextrin (7 : 3, propolis : excipient) by spray dryer process under the same condition used to PMM.

### 2.3. HPLC Analysis

Propolis extracts were evaluated for high pressure liquid chromatography (HPLC) with Shimadzu equipment with CBM-20A controller, LC-20AT quaternary pump, diode array detector SPD-M 20A, and Shimadzu LC version 1.21 SP1 software. For analytical running, Shimadzu Shim-Pack CLC-ODS (M) column was used (4.6 mm × 250 mm, with particle diameter of 5 *μ*m with porous diameter of 100 Å). To evaluate phenolic derivatives, the eluent solution consisted of methanol and acidic water with formic acid (0.1% w/w), 20–95%, 77 minutes of running, and 0.8 mL/min of flow [15]. The detection was at 275 nm. PEE, PWE, PMM, and PSDE (*n* = 3) were individually diluted with methanol/water and homogenized with ultrasound bath. After that, the volume was acidified with formic acid to pH 2.70. After the filtration (0.45 *μ*m), 20 *μ*L was injected in HPLC system.

### 2.4. Strains, Media, and Culture Methods

*C. albicans* strains used were SC5314 (*wild type) *[16], CAI4 (*ura3::imm434/ura3::imm434 iro1/iro1::imm434*) [17], 3153A (wild type) (generously donated by Paul Fidel Jr., Department of Microbiology, Immunology and Parasitology, Louisiana State University Medical Center, New Orleans, LA, USA), *Candida parapsilosis* ATCC 22019, and *C. glabrata* ATCC 90030. *Saccharomyces cerevisiae* BY 4742 was also used. The media used were the complete media YPD agar (2% w/v glucose, 1% w/v yeast extract, 2% w/v peptone, and 2% w/v agar) and YPD liquid medium with the same composition (but without agar).

### 2.5. Viability Determination and Kinetics

*Saccharomyces cerevisiae* BY 4742, *Candida albicans* SC5314, *C. albicans* 3153A, *C. parapsilosis* ATCC 22019, *C. albicans* CAI4, and *C. glabrata* ATCC 90030 were incubated in YPD liquid medium for 16 hours (stationary phase growth) at 30°C. After this period, 1 × 10^6^ cells/mL were inoculated into 30 mL of YPD liquid in 125 mL erlenmeyer flasks. Cells were treated with 0.125, 0.250, 0.500, 0.75, and 1.00% of propolis dry matter from PEE, PWE, PMM, and PSDE. For negative controls, it was employed ethanol at the same amount present in each propolis concentration (PEE possess 55% w/w of ethanol) or phosphate buffer solution (pH 7.4). The samples were maintained in the medium for 1, 2, 4, 6, 8, 12, and 24 hours. The erlenmeyer flasks were kept in agitation of 180 rpm at 30°C for 24 hours. After these periods of treatment, in order to verify their viability, tenfold serial dilution of these cells was plated on Petri dishes containing solid YPD medium. They were incubated at 30°C for 24 hours. Besides this procedure, a sample of treatments with 1% of propolis, with 12 and 24 hs of incubation (*n* = 3), was dispersed in YPD medium, incubated at 30°C for 24 hours, in order to plate and verify the viability of the strains in comparison with the viability of the strains treated with the controls (alcoholic solution or PBS). All the colonies found in each plate were counted. Controls correspond to 100% of viability, and then, the number of colonies found in the treatment groups was compared with the controls in order to determine the percentage of viability in the presence of propolis extracts. 

In order to investigate the role of some phenolic compounds, caffeoyl derivatives and the terpenoid *trans*-nerolidol present in propolis extracts in the antifungal action, the isolated substances: caffeic, *p*-coumaric, and cinnamic acids, aromadendrin, isossakuranetin, Artepillin C and 3,4; 4,5; 3,5 and 3,4,5 caffeoylquinic acids and finally, *trans*-nerolidol were studied in *C. albicans* SC5314. For this purpose, YPD liquid medium was used in 96 vessel plaque containing 10^4^ cells/vessel of *C. albicans*, and each standard was diluted in DMSO and was evaluated at 1, 2, 3, 4, 5, 12.5, 50, 75, 100, 200, and 300 *μ*g/vessel. To control the experiment, it was also used (i) the inoculum group, (ii) DMSO and (iii) fluconazole treatment. The plaque was incubated at 30°C for 24 hours. The evaluation involved visual inspection of “pellet” formation and the evaluation of viability. For viability assessment, 5 uL of each vessel content was put in YPD complete medium at 30°C for 24 hs.

### 2.6. Minimum Fungicidal Concentration (MFC)

The broth dilution method recommended by the Clinical and Laboratory Standards Institute (document M27-A3, CLSI, 2008) [18] was used in this study, with some modifications, in order to determine minimum fungicidal concentration of the samples. In this study, the following microorganisms were used: *C. albicans* SC5314, *C. parapsilosis* ATCC 22019, and *C. glabrata* ATCC 90030.

Because of the turbidity that occurred in test broth when propolis extracts were diluted in the culture medium, it was not possible to determine the minimum inhibitory concentration (MIC). Therefore, the antifungal activity of the samples was assessed by means of the minimum fungicidal concentration (MFC), which was determined by subculturing 20 *μ*L aliquots from each tube of the broth dilution series onto Potato Dextrose Agar (Difco, Detroit, MI, USA). The plates were incubated at 35°C aerobically for 48 h. After the incubation period, the MFC was determined. It was defined as the lowest concentration of the sample required to kill the microorganism being tested.

### 2.7. Propolis Effects during Morphologic Transition

Aiming to evaluate *C. albicans* sensitivity to propolis (PMM) (0.5% subinhibitory concentration) in different morphologic types, SC5314 strain was inoculated in YPD medium for 16 hours at 30°C under agitation. After that, cells were counted, and 2 × 10^7^ cells/mL were incubated for 4 hours under the same previous conditions (yeast), in YPD at 37°C (to induce pseudo-hyphae), and in YPD liquid with bovine fetal serum (20%) at 37°C. To control the experiment, phosphate buffer was used in substitution of PMM for the same time, 6 hours. To evaluate the efficiency of morphologic conditions, the sample was evaluated by microscopy. After treatments, a tenfold dilution was done from 10^6^ cells/mL and “drop out” under YPD complete medium. Plates were incubated for 24 hours at 30°C.

### 2.8. Gel Preparation

#### 2.8.1. Propolis Based Carbopol 940 Gel (CP1%)

Carbopol (1% w/w) was dispersed in water previously conserved (potassium sorbate 0.1% and EDTA 0.01%), and the polymer was maintained under hydration for 24 hs. Then, the mixture of propolis extract (1%), Melaleuca (*Melaleuca alternifolia*), sweet birch (*Betula lenta*), Mentha (*Mentha spicata*) and rosemary (*Rosmarinus officinalis*) essential oils, and propylene glycol was added into the dispersion previously obtained with stirring. Under stirring, triethanolamine was used to adjust pH of the preparation.

#### 2.8.2. Propolis Based Poloxamer 407 Gel with Carbopol 940 (PP1%)

Initially, the co-polymer Poloxamer 407 (Lutrol F127) (13% w/w) was dispersed in water and the polymer was maintained under hydration for 24 hours at 5°C. Meanwhile, Carbopol (1% w/w) was dispersed in water previously conserved (potassium sorbate 0.1% and EDTA 0.01%) and was maintained under hydration for 24 hours (phase B). Then, under stirring, the mixture of propolis extract (1%), *Melaleuca* (*M. alternifolia*), sweet birch (*B. lenta*), *M. spicata* and rosemary (*R. officinalis*) essential oils, and propylene glycol was added gradually into the poloxamer dispersion previous obtained (phase A). Afterwards, carbopol 940 dispersion was mixed in phase A. Under stirring, triethanolamine was used to adjust pH and confer the jellification of the preparation around pH 6.0.

#### 2.8.3. Propolis Alginate with Pectin (AlP1%)

Sodium alginate (4%) was dispersed in water and maintained under hydration for 2 hours. Then, the mixture of pectin (2%), propolis extract (1%), *Melaleuca* (*M. alternifolia*), sweet birch (*B. lenta*), *M. spicata* and rosemary (*R. officinalis*) essential oils, and propylene glycol was added gradually into the dispersion previously obtained with stirring to complete homogenization. 

#### 2.8.4. Propolis Based Chitosan Gel with Natrosol (ChP1%)

To dissolve and jellify natrosol (4.0%), the sample was dispersed in water under warming at about 70°C. After that, the preparation was cooled (A). Meanwhile, chitosan (1.5%), propolis extract (1%), propylene glycol, potassium sorbate, EDTA, *Melaleuca* (*M. alternifolia*), sweet birch (*B. lenta*), and *M. spicata* and rosemary (*R. officinalis*) essential oils were weighted and blended (B). Phase B was included in phase A under stirring. Acetic acid was dripped, and gel formation could be observed.

For all previous preparations, controls were prepared using alcoholic solution (55% w/w) in substitution of propolis and were identified as the controls: CC-(carbopol), PC-(poloxamer), AlC-(alginate) and ChC- (chitosan). Moreover, none of the controls possess essential oils.

### 2.9. Flow Rheology

For this study, a R/S Plus Rheometer Brookfield v.9.0 was used, coupled with a Peltier system with Software Brookfield, RHEO 2000, version 2.8. The condition of study was 37.5°C to resemble body temperature, and plate P25 was used (P25 module), because it is the analysis system for semisolids. As parameter of analysis, a race of 120 seconds was done, with upward curve from *t*0 to *t*60 s and descendant from *t*60 s to *t*120 s, obtaining values of shear stress, shear rate, and viscosity every 2 seconds. Assays were done in triplicate, and statistical analysis using software Brookfield, based on calculations by Ostwald Law and Prism 5.0 software.

### 2.10. Mucoadhesion Tests

To mucoadhesion studies, the reproductive system of the cow was removed immediately after sacrificing the animal in the slaughterhouse Olhos D'água (Ribeirão Preto, SP, Brazil) and vaginal mucosa removed with scalpel and surgical scissors and immediately frozen at −10°C. On the day of the experiment, the mucosa was thawed and cleaned using 0.9% NaCl solution at 25°C, cut into disks of 1 cm diameter, and glued with cyanoacrylate glue (SuperBonder) in a holder made of Plexiglass holder. The test was done by lowering the load cell to contact with the mucosa for a time of 30 seconds and tensile strength of 0.5 N at a rate of 0.5 mm/min. Triplicates were performed for each gel evaluated and statistical analysis with Prism 5.0 software. In this study, the machine Emic DL 2000 was used with a load cell 19 Trd, with the program Tesc, version 3.01. 

### 2.11. Candidiasis Vaginal “*In Vivo*” Model

This experiment was approved by the Animal Experimentation Ethics Committee of the Faculty of Pharmaceutical Sciences, University of São Paulo (FCFRP/USP), Ribeirão Preto, SP, Brazil. To this study, female Balb/c mice with 7-8 weeks of age, weighing approximately from 20 to 22 g, were used in this study. 

This protocol was performed as described by Yano and Fidel Jr. [19] with some modifications. Thus, to induce vaginal candidiasis in mice Balb/c animals, it was necessary to simulate pseudoestrus condition for optimal *Candida* colonization. For that, 0.3 mg of estradiol valerate (17 *β*-estradiol valerate) previously dissolved in 100 uL of castor oil (Sigma-Aldrich) was injected in dorsal area of each animal, followed by a massage to disperse the suspension, 72 hours before infection. Pseudoestrus condition was maintained weekly with the application of the hormone, a condition necessary to guarantee the results of the protocol. For the infection, 20 *μ*L of the *C. albicans* strain suspension was inoculated by inserting the pipette tip about 5 mm deep into the vaginal lumen.

#### 2.11.1. *Candida albicans* Suspension

*C. albicans* strain was incubated for 24 hours in YPD complete medium at 30°C. Then, one colony was dispersed in liquid YPD medium, under agitation, at 30°C for 16 hours. The suspension was washed with PBS until a cleaner suspension was obtained; then, after centrifugation, the pellet was suspended in PBS, counted with Neubauer chamber and diluted to 2.5 × 10^6^ cell/mL. The suspension of *Candida albicans* was intravaginally inoculated (20 *μ*L) [19].

#### 2.11.2. Standardization of the Protocol

 In order to define the best strain and time after inoculation of *C. albicans* to start treatments, the fungal burden and histological slices of the animals after 48 and 72 hours after inoculation of *C. albicans* was investigated. After that, the protocol with the time predefined in the last protocol was used to compare between *C. albicans* SC5314 and 3153A.

#### 2.11.3. Treatments Protocol

The groups of animals were divided into six (*n* = 10): (i) control (animals infected without treatment), (ii) treated with CPb (carbopol gel base), (iii) treated with PPb (poloxamer gel base), (iv) treated with CP1% (propolis 1% carbopol gel), (v) treated with PP1% (propolis 1% poloxamer gel), and finally (vi) treated with clotrimazol cream (Neo Química). Each group was treated every 12 hours with 60 uL of each product (except for group one—without treatment), during 7 (seven) and 10 (ten) days. After treatments, the evaluation of the animals was done with the culture of intravaginal lavage (100 uL of PBS) in each time and with the histological evaluation of vaginal mucosa of one animal of each group. Then, the animals were supervised during the 10 days after stopping of the treatment and re-evaluated with the culture of intravaginal lavage and the histological slices of the vaginal mucosa.

One or two animals per group was euthanized (cervical dislocation) with 7 and 10 days of treatment and in the conclusion of the protocol (10 days after the last treatment) for vaginal mucous removal and histological slices preparation. The specimens were removed and fixed for 24 hours in 3.7% formaldehyde—PBS. Samples were washed several times in 70% alcohol before dehydration in a series of alcohol solutions of increasing concentrations. Finally, the samples were diaphonized in xylol and embedded in paraffin. For each sample, sequential 5 *μ*m thick sections were collected on glass slides and stained with Gomori methenamine silver (GMS) or hematoxylin and eosin (H&E) stain. Briefly, sections were deparaffinized, oxidized with 4% chromic acid, stained with methenamine silver solution, and counterstained with picric acid. For H&E staining, sections were deparaffinized and stained first with hematoxylin and then with eosin. All stained slides were immediately washed, preserved with mounting medium, and sealed with a coverslip. Next, they were analyzed and photographed under a microscope (Jenaval-Zeiss) coupled to a digital camera (Leica DFC425). The parameters analyzed were descriptive and examined by a single researcher who was blinded to the analysis of the groups. The parameters analyzed were inflammatory reaction and *C. albicans* infection.

### 2.12. Statistical Analysis

The analysis of variance ANOVA (one way) and Bonferroni multiple comparison test was performed with a level of significance of 5% or by Student's *t*-test (*α* = 0.05) according to the protocol used. Statistical analysis was done using Prism 4 (Graph Pad).

## 3. Results

### 3.1. Chemical Characterization

**Figure 1:**
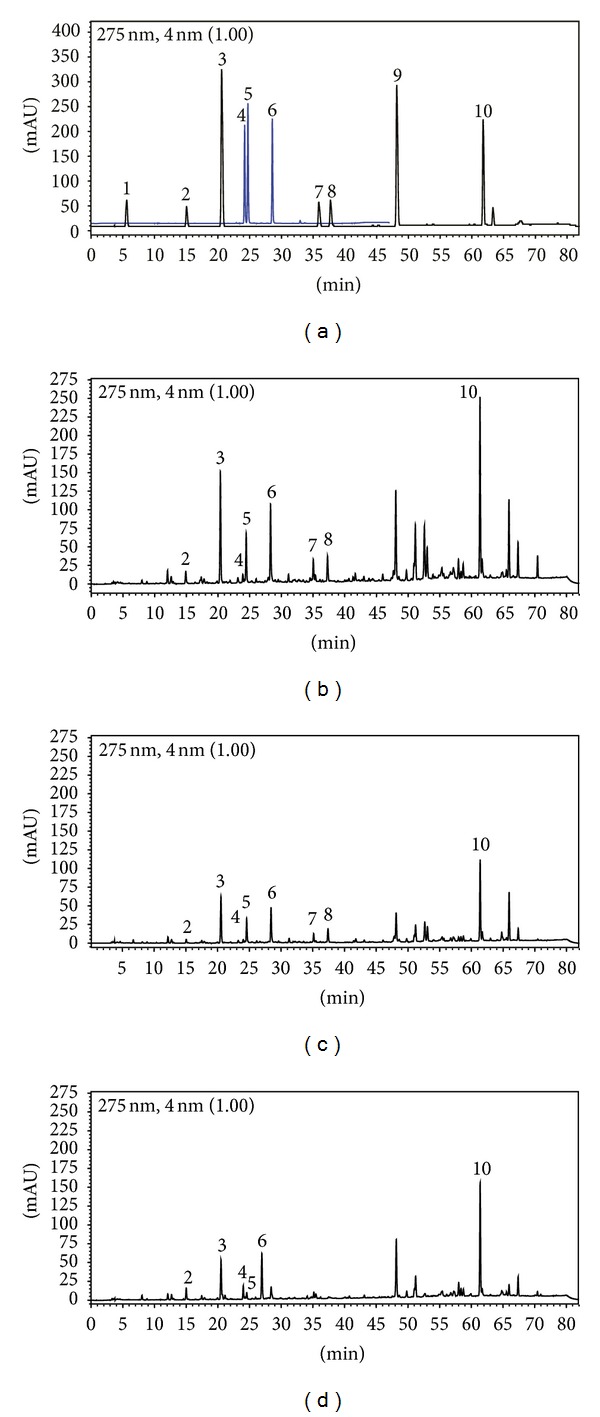
Chemical fingerprint of (a) standards used: (1) internal standard : gallic acid, (2) caffeic acid, (3) *p*-coumaric acid, (4) 3,4-dicaffeoylquinic acid, (5) 3,5-dicaffeoylquinic acid, (6) 4,5-dicaffeoylquinic acid, (7) cinnamic acid, (8) aromadendrin, (9) isosakuranetin, and (10) Artepillin C, and of the extracts evaluated, PEE (b), PMM (c), and PSDE (d) (*n* = 3). The chromatograms were plotted at 275 nm, using the RP-HPLC, C18 (Shim-pack, CLC-ODS (M), 25 cm × 4.6) column, and gradient elution with methanol and acidic water (formic acid pH = 2.7).

**Figure 2:**
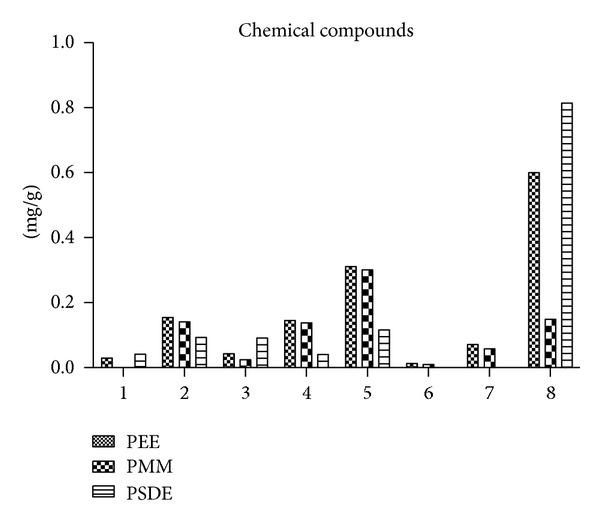
Chemical results obtained for PEE, PMM, and PSDE after normalization of the extracts to 1% of propolis dry matter (mg/g). The results originated from the medium previously presented in Table 1; however, in order to pollute the figure less, the standard deviations were suppressed. (1) Caffeic acid; (2) *p*-coumaric acid; (3) 3,4-DCQ acid; (4) 3,5-DCQ acid; (5) 4,5-DCQ acid, (6) cinnamic acid; (7) aromadendrin, and (8) artepillin C.

Although the standardization of propolis extract (EPP-AF) was previously shown by Berretta et al. [13], the HPLC fingerprints of PMM and PSDE extracts were also performed (Figure 1) since these extraction processes involve several steps, such as temperature, filtration/purification, dryness, and others. These steps could provide different chemical composition for each extract differing from the same EEP standardized propolis batch. The results of each extract are presented in Table 1, and the results normalized to the same propolis concentration (1%) are shown in Figure 2. It is possible to detect that the preparation process led to the absence of cinnamic acid and aromadendrin in PSDE and the absence of caffeic acid in PMM (lower than quantification limit). It was observed that PEE and PMM presented few differences considering most of the standards evaluated (normalized by propolis dry matter); however, artepillin C presented a reduction of about 25% of the content present in PEE. Interestingly, PSDE showed expressive values to artepillin C, 0.816 mg/g. Caffeoyl quinic acid derivatives were also evaluated since a recent work attributed antiviral activity to these compounds [20]. Results are presented in Figure 1 and Table 1. It is possible to observe similarities in PEE and PMM, corroborating the results obtained with phenolic derivatives. It is known that propolis aqueous extract usually presents caffeoyl quinic acid derivatives in their composition [20–22]; however, this is not the case in the present work since PWE was obtained from PEE and the extraction solvent was not water.

Taken together, the chemical characterization results showed that PEE and PMM are similar (*P* > 0.05) considering the standards investigated, while PSDE is different from PEE and PMM (*P* < 0.05).

### 3.2. Antifungal Comparison of Propolis Extracts in “*In Vitro*” Models

**Figure 3:**
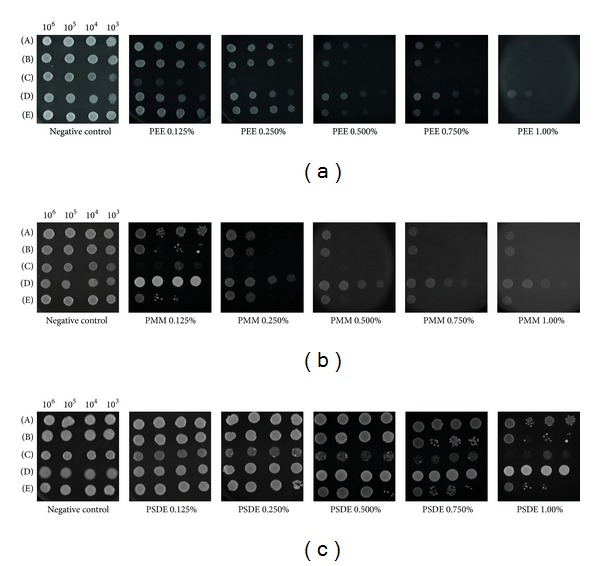
Antimicrobial activity of different propolis extracts evaluated, PEE, PMM, and PSDE, using *C. albicans* SC5314 (A), CAI4 (B), *S. cerevisiae* (C), *C. parapsilosis* (D), and *C. glabrata* (E) strains, with 0.125%, 0.250%, 0.50%, 0.75%, and 1.0%. The controls of the experiment were the strain in YPD medium, alcoholic solution in the same concentration of propolis extract, and phosphate buffer. The strains were evaluated in YPD complete medium with propolis, at 30°C, 24 hs.

In order to compare the antifungal activities of each extract, PWE, PEE, PMM and PSDE (0.125, 0.25, and 0.50% of propolis) were evaluated against *Saccharomyces cerevisiae* and *C. albicans* (data not shown). The experimental conditions, such as propolis subinhibitory concentration, appropriate growth phase of microorganism, and others, were previously defined by de Castro et al. [23]. *S*.* cerevisiae* was the less tolerant strain to all tested extracts. The results showed that PEE was the most potent for both microorganisms followed by PWE, PMM, and PSDE. The MFC was determined since it was not possible to establish the MIC determination due to propolis turbidity. The MFC value found was 7.0 mg/mL for PEE, considering the microorganisms *C. albicans* and *C. parapsilosis*, and 14.0 mg/mL for *C. glabrata*. The other results can be seen in Table 2, where it is evident the better efficacy of PEE compared to the other extracts. In this assay, the MFC value of PWE was very low, and consequently, this extract was not investigated in other steps, including chemical characterization. After the first trial, extracts were evaluated in propolis concentrations from 0.25 to 1.0% against *S. cerevisiae*, *C. albicans* SC5314 and CAI4 strains, *C. parapsilosis*, and *C. glabrata* (Figure 3). The results demonstrated that PEE was more effective than PMM and PSDE considering all strains tested (Figure 3). *S. cerevisiae* followed by *C. albicans* were more sensitive than the other strains, while *C. parapsilosis* was the most resistant strain studied (Figure 3). These results were corroborated with the MFC values presented in Table 2, for PEE, PMM, and PSDE.

**Figure 4:**
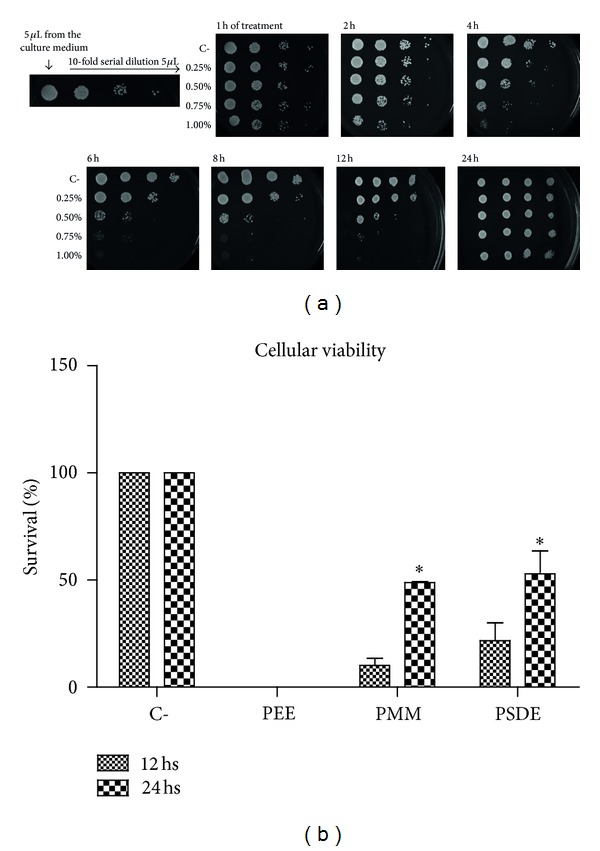
(a) Antimicrobial activity of PMM using *C. albicans* SC5314 with 0.250%, 0.50%, 0.75%, and 1.0% of propolis with 1, 2, 4, 6, 8, 12, and 24 hours of treatments (30°C, 24 hs under agitation). The controls of the experiment were the strain in YPD medium and PBS. After the treatments, 5 *μ*L and subsequently tenfold dilutions were applied in YPD complete medium with propolis, at 30°C, 24 hs; (b) graphic presentation of cellular viability *of C. albicans* treated during 12 and 24 hours with PEE, PMM, and PSDE 1% (*n* = 3).

To evaluate *C. albicans* cell viability upon propolis exposure, PMM was incubated for 1, 2, 4, 6, 8, 12, and 24 hours with 10^6^ cells/mL. The results showed that the best growth inhibition occurred at 6 and 8 hours of propolis exposure (Figure 4(a)). Unexpectedly, at 24 hs incubation with PMM, *C. albicans* grew at the same density as the control (Figure 4(a)). Thus, *C. albicans* viability was assessed for all different propolis extracts incubated for 12 and 24 hs, and accordingly, fungicide and fungistatic actions of the extracts were evaluated, considering the results of samples plated and incubated for 24 hs at 30°C. Viability results with 1% of propolis showed that PEE has fungicide effects, while PMM and PSDE have fungistatic action at this concentration, that is, 10 mg/mL, and also considering the incubation time of 12 and 24 hs (Figure 4(b)). Taken into consideration the results observed, the chemical changes among the extracts emerge as the possible responsible for these growth differences. Thus, volatile substances were researched by Gas Chromatography (GC) since both PMM and PSDE were obtained with high pressure and relatively high temperatures (spray drying process), and consequently, volatile substances could be different. The results demonstrated that PEE presented innumerous volatile compounds, specially *trans*-nerolidol and spathulenol (J. P. De Sousa & J. K. Bastos, data not published), while PMM and PSDE did not show any volatile substances in the analytical running used (data not shown). Although these volatile substances were different, these isolated compounds were investigated and the results showed that none of them demonstrated inhibitory action up to 100 *μ*g/vessel, except for fluconazole that exhibited this effect upon 10 *μ*g/vessel.

**Figure 5:**
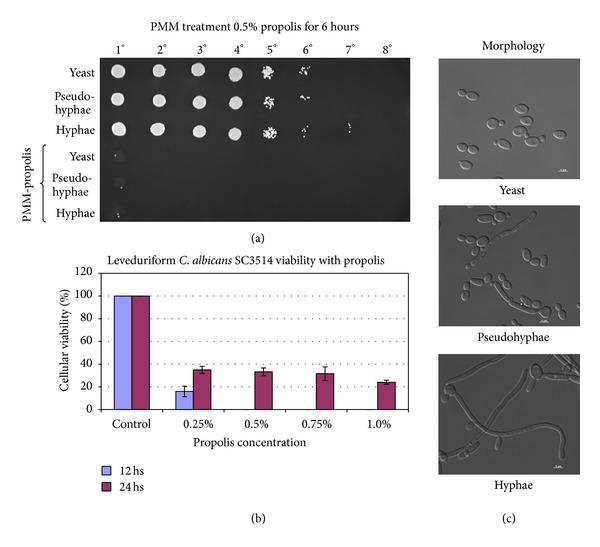
(a) Antimicrobial activity of PMM using *C. albicans* SC5314 (yeast, pseudohyphae, and hyphae) with 0.50% of propolis with 6 hours of treatments (30°C, 24 hs under agitation). The controls of the experiment were the strain in YPD medium and PBS. After the treatments, 5 *μ*L and subsequently tenfold dilutions were applied in YPD complete medium with propolis, at 30°C, 24 hs; (b) graphic presentation of cellular viability of *C. albicans* treated during 12 and 24 hours with 0.25, 0.50, 0.75, and 1.0% of propolis (PMM) (*n* = 3); (c) microscopy of the strains used during protocol, yeasts, pseudohyphae, and hyphae.

The dimorphic transition is an important step for *C. albicans* pathogenicity, and the growth inhibition of different morphological forms like hyphae, pseudohyphae, and yeast (Figures 5(a) and 5(c)) was studied in the presence of propolis (PMM).  Figure 5(a) shows that the treatment of *C. albicans* during 6 hours with PMM (0.5% propolis) is effective for all transition stages, and this fact is dependent on PMM concentrations (Figure 5(b)).

### 3.3. Propolis Gels Evaluation: Antifungal, Rheology and Mucoadhesion in *In Vitro* Models

**Scheme 1:**
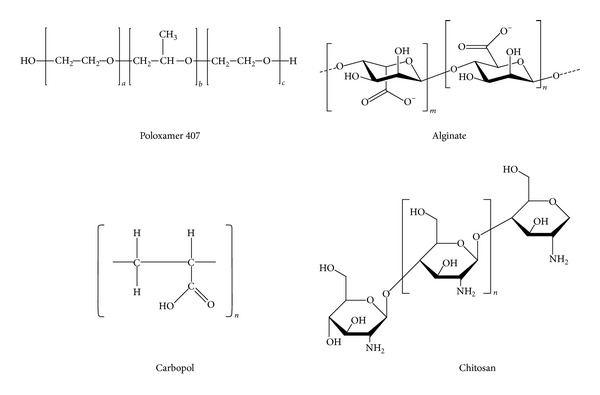


Among all available polymers, we have chosen for the present study chitosan, sodium alginate, carbopol 940, and poloxamer 407 Scheme 1. Chitosan, a cationic chain known to be an excellent material for drug preparation, is a plentiful natural biopolymer, nontoxic, biocompatible, and biodegradable [24]. Alginic acids or the salt forms, sodium alginate, are food ingredients and they have been used as additives for drug preparation due to their safety to oral administration and for controlling drug release and biocompatibility [25]. Poloxamer 407, a nonionic copolymer of poly(oxyethylene)-poly(oxypropylene)-poly(oxyethylene), has been studied as a potential base for thermosensitive hydrogels. It can carry sufficient drug and shows good water solubility, tolerability, biodegradability, nontoxicity, and controlled release [26]. Carbopol 940 is a cross-linked polyacrylate polymer of anionic character. It is an extremely efficient rheology modifier able to provide high viscosity and forms sparkling clear water or hydroalcoholic gels and creams. All formulations were evaluated for *in vitro* effectiveness dissolving each one in YPD top agar medium with propolis at 0.125%, 0.25%, 0.50%, 0.75%, and 1.0%. Subsequently, tenfold serial dilution from *S. cerevisiae*, *C. albicans*, *C. parapsilosis*, and *C. glabrata* cells was plated in each propolis concentration. It was observed that all formulations at concentrations higher than 0.5% propolis demonstrated similar results to every tested strain (data not shown). At concentrations lower than 0.5% of propolis, *S. cerevisiae* was the least propolis-tolerant microorganism. After demonstrating the *in vitro* effectiveness, formulations were physicochemically characterized aiming to perform the *in vivo* evaluation. 

Rheological studies are important to develop semisolid preparations, especially when certain characteristics should be present, such as easiness in product removal from packaging and application, adequate spreading, and smooth texture on the application site [27]. Some important features as spreadability and retention characteristics are essential to clinical outcome of vaginal semisolids, particularly those with contraceptives and microbicidal activity [28]. 

**Figure 6:**
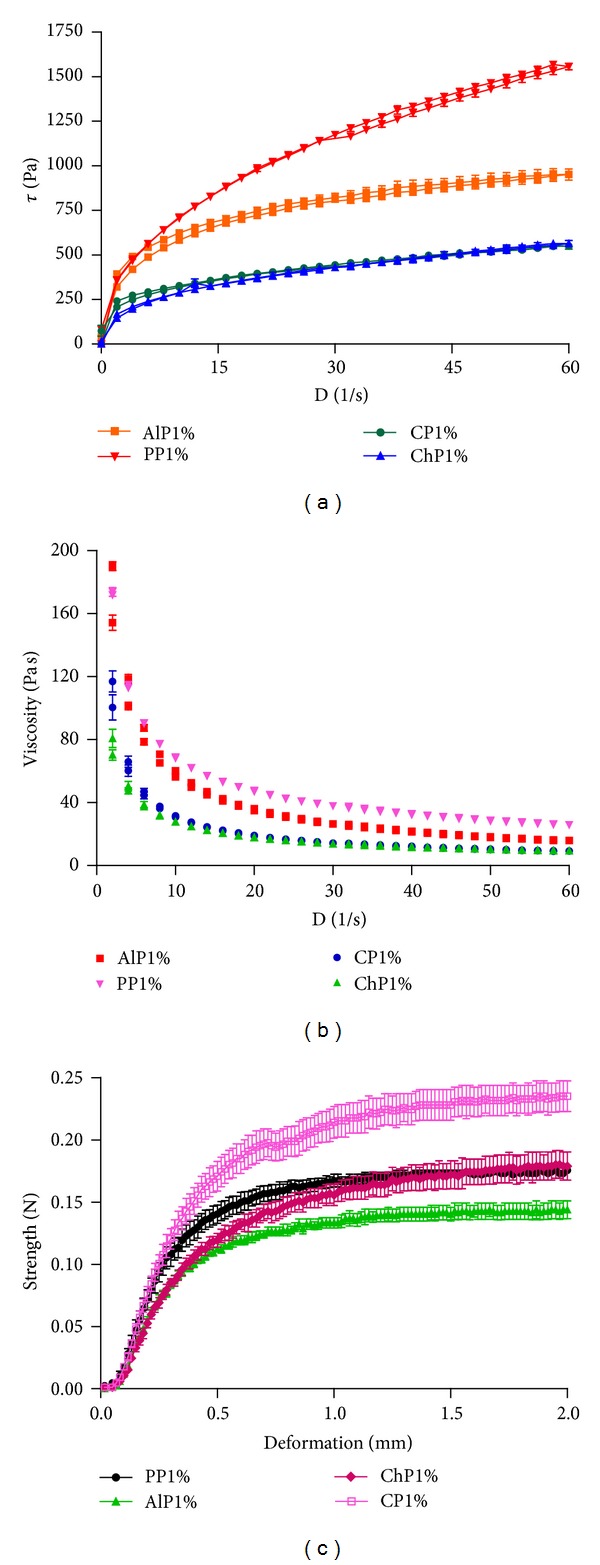
Rheograms of formulations were (a) presents shear stress, (b) viscosity, and (c) mucoadhesive results.

Flow properties of vaginal formulations determine the ease of product administration into the mucosa membrane and the (time-dependent) recovery of the product following administration. In this context, Newtonian and non-Newtonian behaviors can be seen. In continuous shear rheometry study, all formulations exhibited shear-thinning behavior (pseudoplastic flow) with low degrees of thixotropy (Figure 6(a)). The decreases in the non-Newtonian viscosity as a function of increasing shear rate were most appropriately mathematically modelled using the Power law (Oswald-de Waele) model where the flow behavior index (*n*) was determined [29]. Although Figure 6(a) clearly shows that all preparations presented pseudoplastic behavior, its magnitude can only be clearly observed by the results shown in Table 3. It is known that the Newtonian characteristics are as higher as the value found for the material is closer to 1 and, consequently, as the value is lower than 1, the more pseudoplastic the material will be [28]. Pseudoplasticity of poloxamer and chitosan based gels was modified by propolis presence (Table 3), while no changes were observed to sodium alginate and carbopol gels (*P* > 0.05). The flow indexes values were 0.4455 ± 0.0017, 0.2554 ± 0.0095, 0.2607 ± 0.0073, and 0.3674 ± 0.007 for PP1%, AlP1%, CP1%, and ChP1%, respectively (Table 3). As the flow index values were all below 1, graphic representation was confirmed showing that all formulations presented pseudoplastic behavior [30], which is typical of the polymeric system [28]. There were significant differences (*P* < 0.05) among all formulations, except when comparing alginate and carbopol (*P* > 0.05). 

Considering viscosity results, Figure 6(b) shows that carbopol and chitosan hydrogels are less viscous preparations, while poloxamer is the most viscous one. This is an interesting property that can help to retain the preparation into vaginal mucosa. Although poloxamer presented the worst pseudoplastic results, it is important to consider that this gel base has properties, such as thermoreversible behavior with increased viscosity in corporeal temperature [31, 32], and usually this composition offers a delivery system, information that can be interesting in the pathology in study.

An essential property that governs the clinical performance of mucosal gels is the ability to adhere to the host epithelium and provide residency during the therapeutic period. Hence, mucoadhesive formulations of limited viscosity have been employed for this purpose [33]. The mucoadhesive bond strength was examined using a previously reported test in which cow vaginal mucosa was employed, and the force required to partially separate the mucosa disc from the surface of the formulation is determined. The ability of a vaginally applied formulation to adhere to the vaginal epithelium is essential to maximize its residency and thereby clinical performance [34]. It has been reported that the establishment of a mucoadhesive bond between polymeric components and a biological substrate may be influenced by the surface of the biological substrate, surface of the bioadhesive layer and interfacial layer between the two [35]. Assuming that the surface of the mucosa in each experiment is similar, slight differences occurring in the mucoadhesive ability of the gel formulations may be attributed to the formulation surface effects. The results showed that propolis inclusion increased mucoadhesion of CP1% and PP1%, a fact not observed in alginate and chitosan vehicles (data not shown). It was observed that the CP1% based gel demonstrated higher strength (0.24 N), followed by ChP1% (0.18 N) and PP1% (0.17 N). The difference that favors poloxamer is the little time to reach the mucoadhesion stabilization in comparison to chitosan based gel. This could be related to the differences in the polymer chain flexibility, ability to form hydrogen bonds and/or the extent of swelling of polymers.

### 3.4. Propolis Gels: Preclinical Efficacy Evaluation

**Figure 7:**
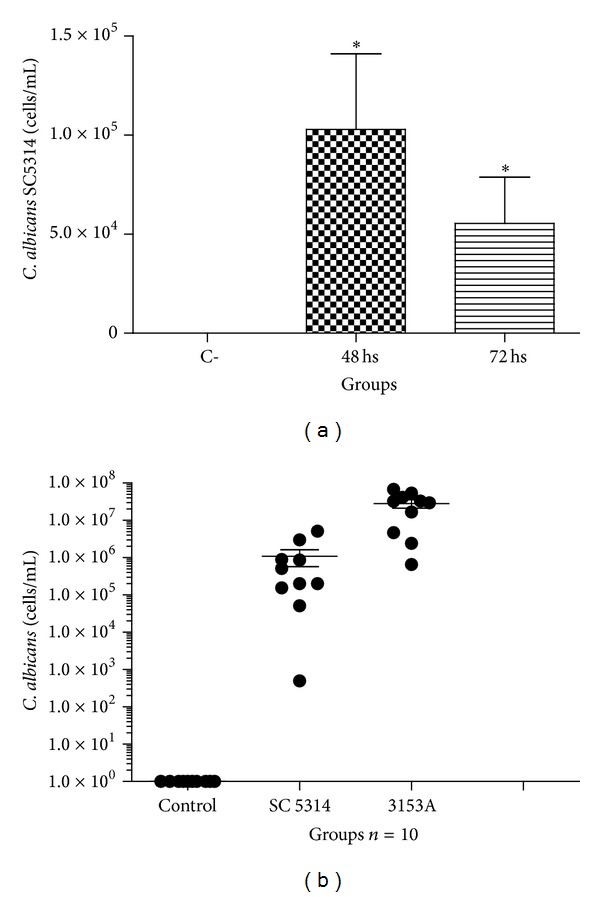
(a) Fungal burden of vaginal mucosa lavage after 48 and 72 hours of inoculation of *C. albicans* SC5314 (20 *μ*L, 2.5 × 10^6^ cells/mL) (YPD complete medium, 30°C, 24 hs); (b) fungal burden of vaginal mucosa lavage after inoculation of *C. albicans* SC5314 and 3153A with 48 hours after inoculation (YPD complete medium, 30°C, 24 hs); (*P < 0.05—*Student's t*-test).

**Figure 8:**
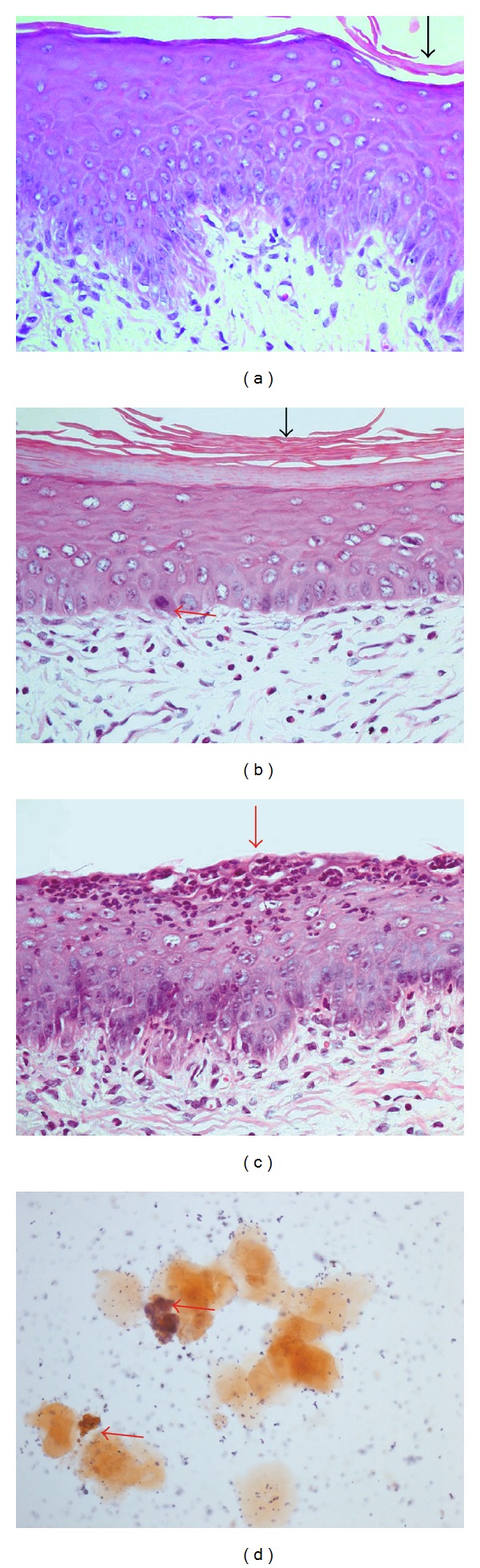
(a) Control group (without infection): vaginal mucosa with normal architecture and normal thickness showing discrete keratin deposition (H&E-40x); (b) *C. albicans* SC5314 infection group: normal architecture with decreased thickness with signs of epithelial regeneration (mitosis) and increased of keratin deposition (H&E-40x); (c) *C. albicans* 3153A infection: normal thickness with precursor cells increase, superficial erosion, and neutrophils influx, without keratin (H&E-40x); (d) neutrophils presence in lavage microscopy (ASD-Naphtol-20x).

The *in vivo* vulvovaginal candidiasis model was established 48 hours after inoculation (Figure 7(a)). In this model, *C. albicans* 3153A strain was more virulent than SC5314 (Figure 7(b)), a fact corroborated by Figures 8(a)–8(d). The histological analysis demonstrated that the control group (Figure 8(a)) displayed normal tissue, while vaginal mucous infected with SC5314 strain showed keratin deposition and decreased thickness, a behavior more characteristic of chronic infection and common in less virulent strains (Figure 8(b)). Finally, Figures 8(c) and 8(d) showed superficial erosion and neutrophil influx, respectively, features characteristic of acute infection.

To evaluate efficacy, two formulations were chosen considering rheology and mucoadhesion results, carbopol and poloxamer gel with propolis extract, the CP1% and PP1%, respectively. Either preparation was applied every 12 hours for 7 and 10 days. As a control, a group did not receive any treatment, and two other groups were treated with carbopol and poloxamer gel base. To compare propolis effects, clotrimazole (10 mg/g) cream (Neo Química, Brazil), a conventional medicine used in this kind of pathology, was used like a positive control.

**Figure 9:**
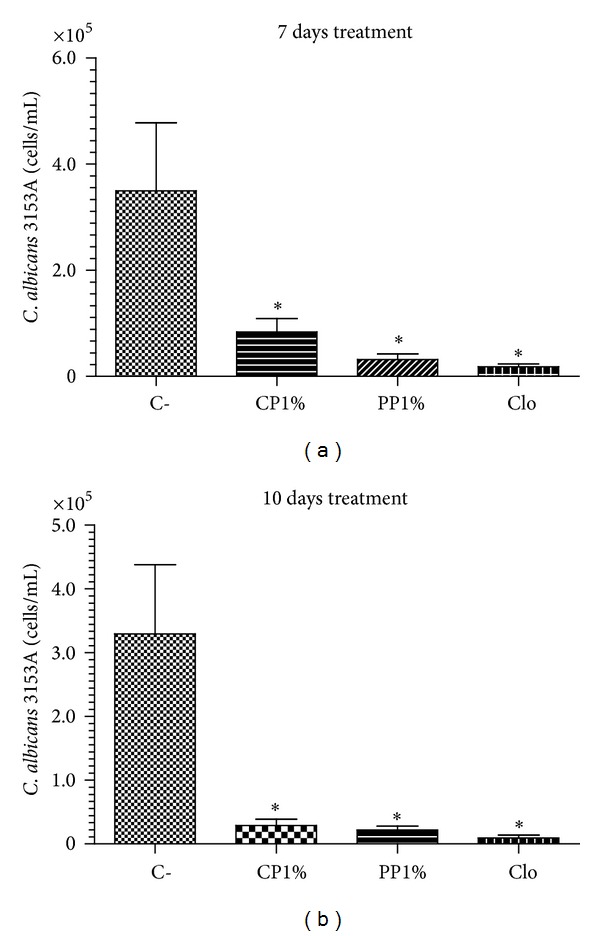
Fungal burden of vaginal mucosa lavage after 7 (a) and 10 days (b) of treatment with CP1%, PP1%, and clotrimazol cream (Neo Química) after infection of *C. albicans* 3153A (20 *μ*L, 2.5 × 10^6^ cells/mL) (YPD complete medium, 30°C, 24 hs). (*P < 0.05—ANOVA *one-way* with Bonferroni's posttest).

Our results showed that 7 days treatments reduced fungal burden in 60.2, 84.8, and 97.2%, for CP1%, PP1%, and clotrimazole, respectively, while with 10 days treatment, fungal burden reduction was 84.2, 89.4, and 97.9%, respectively. Statistical analysis did not show any difference between 7 and 10 days treatment (*P* > 0.05) either using ANOVA one-way or Student's *t*-test with 95 and 99% of confidence interval. All treatments used demonstrated difference with negative control groups (*P* < 0.05), while CP1% and PP1% were similar to clotrimazole cream (*P* > 0.05), for 7 and 10 days of treatment, respectively Figures 9(a) and 9(b). 

**Figure 10:**
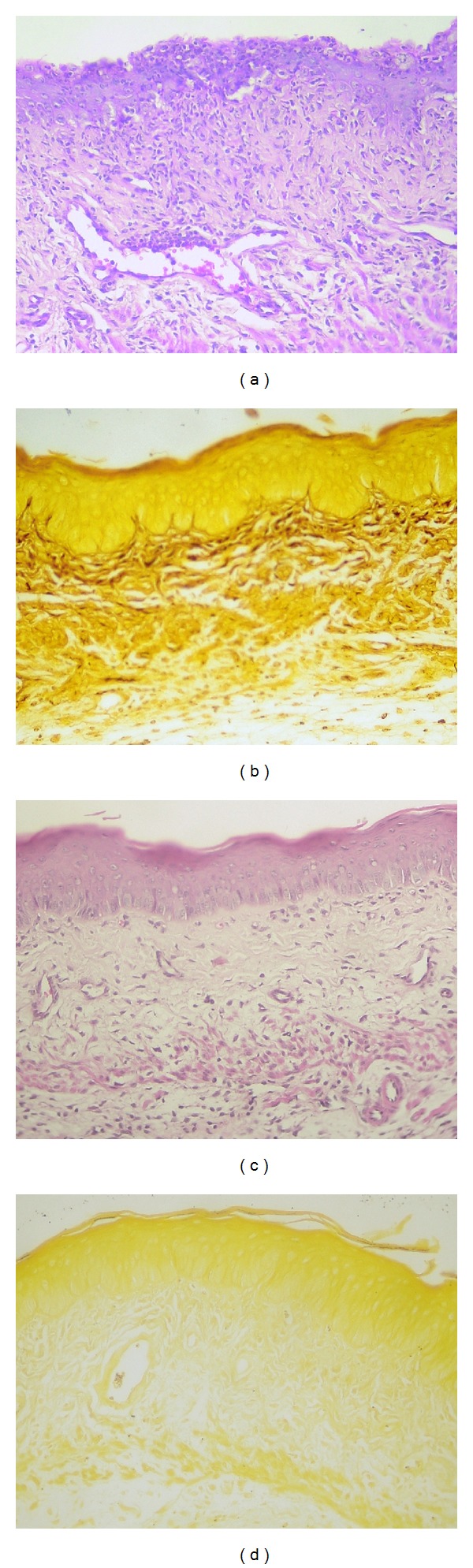
Histological slices of vaginal mucosa with 10 days of infection, showing ((a)-(b)) negative control group and ((c)-(d)) clotrimazole cream treatment (10 days of treatment). Tissular inflammation can be seen in “a” and “c,” with the presence of neutrophils influx (H&E coloration). GMS coloration (b and d) shows *C. albicans* presence in Control group (b), showing the efficacy of the model implementation and absence of *C. albicans* in clotrimazole at this time (d). (×40 magnification).

**Figure 11:**
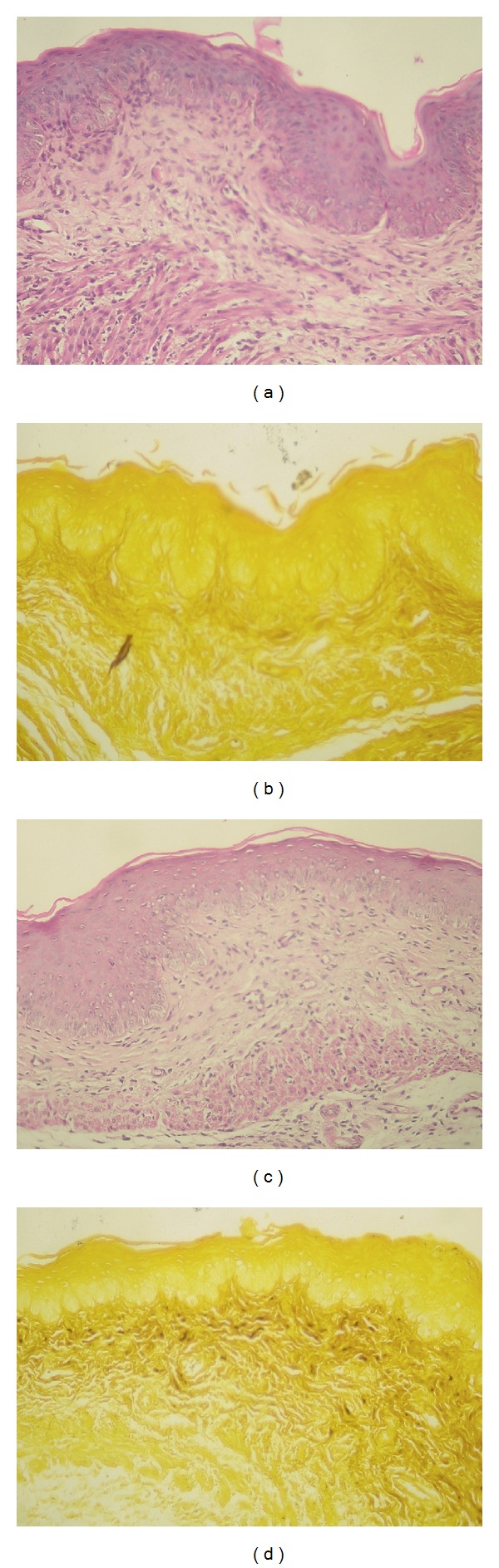
Histological slices of vaginal mucosa after 10 days of infection, showing CP1% group ((a)-(b)) and PP1% ((c)-(d)) treatment during 10 days. Normal tissue can be seen in all pictures (H&E and GMS coloration) and the absence of inflammation and *C. albicans*. (×40 magnification).

The results presented are corroborated by histological analysis upon 10 days treatment (Figures 10(a) and 10(b), the control without any treatment) and Figures 10(c) and 10(d) (clotrimazole group). The results showed that estradiol ministration maintained the pseudoestrus condition and consequent tissue infection considering that *C. albicans* (Figure 10(b)) and inflammation (Figure 10(a)) persisted. In contrast, the clotrimazole treatment presented normal epithelium (Figures 10(c) and 10(d)). The histological analyses of these experiments corroborate fungal burden analysis and in addition demonstrate the absence of irritation or inflammation induced by CP1% and PP1% (Figures 11(a) and 11(c)). Figures 11(a) and 11(c) show the morphological aspects of tissue after 10 days of treatment with CP1% and PP1% where both groups displayed normal architecture and thickness in pseudoestrus phase, with keratin deposition indicating the absence of *C. albicans* infection (Figures 11(b) and 11(d)).

## 4. Discussion

*C. albicans* is a common vaginal inhabitant in humans and is present in their vaginal mucosa during their lives normally not displaying any symptoms or signs of vaginitis and usually with little concentration of yeasts. *C. albicans* can be a commensal or a pathogen into the vagina depending on changes in the host vaginal environment that induce the pathogenic state [1]. Vaginal candidiasis incidence caused by *Candida* non-*albicans* strains has increased in function of unique antifungal dosage forms, low dosage maintenance of the azole posology, and by indiscriminate use of antimicotics [1]. *C. albicans* species represent 85–95% of yeasts isolated from vagina, and considering non-*albicans* strains, the most common is *C. glabrata*, present in 10–20% of the infected women, followed by *C. parapsilosis*. Azoles do not present good results with *C. glabrata* strain [1].

Propolis ethanolic extracts, alone or incorporated in pharmaceutical presentations, are commonly used therapeutically [36]. Considering some disadvantages of this presentation, the present work tried to study other options to use propolis in medicine, such as aqueous, microparticles, and soluble dry extract. Rocha et al. [37] have demonstrated that it is possible to achieve aqueous propolis extract similar in chemical composition to the alcoholic ones, and Bruschi et al. [38, 39] obtained gelatin microparticles with propolis, without taste, strong odour, and without the presence of alcohol. Here, we evaluated PWE, PMM, and PSDE that were developed with higher concentrations of propolis dry matter, that is, around 11%, 50%, and 70% of genuine propolis extract. The antifungal results obtained for PWE were different when compared with the antibacterial results obtained by Rocha et al. [37], and since undesirable results were found, PWE was not considered in the mucoadhesive preparations.

Antifungal effects of propolis *in vitro* have been demonstrated to *C. albicans* by Fernandes Jr. et al. [40], results corroborated by Longhini et al. [41], and the present work. Sawaya et al. [42] who also studied the inhibition of *C. albicans*, *C. tropicalis*, *C. krusei*, *C. parapsilosis*, and others found 20 mg/mL as the MFC for propolis alcoholic extract. This concentration is higher than the values found for PEE, which was 14.0 mg/mL for *C. glabrata*, the most PEE resistant strain, and for PSDE, which was 11.73 mg/mL to *C. albicans* and *C. parapsilosis*. Berretta et al. [13] have shown that different batches of propolis extracts were chemically reproducible, considering phenolic derivatives and also presented anti-inflammatory effects [13, 15, 21, 31]. However, the extracts proposed in this study (PWE, PMM, and PSDE) can show different chemical fingerprints since these kinds of preparations involve several other steps, including high pressure and temperature. This fact was confirmed once a few compounds are absent in some extracts, especially cinnamic acid and aromadendrin in PSDE, and caffeic acid in PMM. This possibly occurred due to the temperature or pressure for PMM and PSDE, necessary steps for the preparation of the pharmaceutics presentations.

Propolis, like other natural products, exert a biologic action through synergic effect between numerous constituents, and the results presented corroborate this information since none of the constituents studied presented anti-*Candida* action up to 100 *μ*g/vessel. Based on the results obtained here for PEE (fungicide), PMM, and PSDE (fungistatic), it is important to consider the volatile compounds of propolis for the better action of propolis extracts, since the difference in PEE and the other extracts by GC analysis was so outstanding. Considering the inherent lipophilic characteristic of terpenoids, they show affinity and partition with biological membranes, where its presence can substantially modify their structural and functional properties [43]. *trans*-Nerolidol is a known compound that increases bacterial plasmatic membrane permeability (*S. aureus* and *E. coli*) [44] and acts in inhibiting the dimorphic transition of *C. albicans* [45]. Brehm-Stecher and Johnson [44] showed that sesquiterpenoids can break the normal barrier of cellular membranes of bacteria increasing the uptake of exogenous compounds to intracellular medium, such as ethidium bromide and antibiotics. This effect was more pronounced in Gram-positive bacteria possibly because they lack additional barriers to the external membrane, for example, Gram-negative bacteria. Brehm-Stecher and Johnson [44] evaluated the ability of nerolidol, farnesol, bisabolol, and apritone sesquiterpenoids to increase the bacterial permeability and the susceptibility of exogenous antimicrobial compounds uptake. These authors demonstrated that sesquiterpenoids promoted intracellular accumulation of ethidium bromide, a nucleic acid marker impermeable to cellular membrane, in *Lactobacillus fermentum* living cells, suggesting that the increase of permeability was a result of cytoplasmatic membrane disorder. Hornby et al. [45] showed that nerolidol and farnesol prevented dimorphic transition of *C. albicans* from yeast to mycelial form. Considering that hyphae are associated with a pathogenic situation, the inhibition of transition can be a nonlethal way to control this pathogen.

Due to their low cost, ease of manufacture, and precedence of use in the topical administration of drugs, conventional gel systems are commonly employed to administer drugs via the vaginal route, mainly for the treatment of vaginal infection, contraception, and hormone replacement therapy. More recently, gel-based formulations are being widely developed for sustained delivery of HIV microbicides and mucosal vaccines. The retention of vaginal gel formulations is fundamental to the improvement of clinical performance. Poor vaginal retention of conventional gel formulations represents a significant challenge for those clinical indications where sustained delivery would enhance efficacy [11]. Flow rheological characterization offered important information considering stability and spreadability, Newtonian and non-Newtonian behavior, thixotropy and viscosity, and important points to consider when developing a topical medicine. It is known that a pseudoplastic material can break down for easy spreading, and the applied film can gain viscosity instantaneously to resist running, a fact not observed with Newtonian fluids independent of the time. Pseudoplasticity is commonly observed in polymeric semisolid preparations, such as skin moisturizing, vaginal microbicides, sunscreens, for example, [28, 29]. Moreover, Newtonian fluids, that is, those whose viscosity is similar independently of shear rate where viscosity is measured, are interesting in developing spermicides [28] or massage fluids, such as mineral oil. Besides therapeutical application, rheological information possibly predicts flowing easiness of the package, intravaginal application, and retention therein, and associated with the viscosity and mucoadhesion results, it is possible to suggest *in situ* behavior. Considering rheological data, our results indicate that CP1% and PP1% were the most promising formulations because of their pseudoplasticity and the viscosity, respectively. The nonlinear responses to shear stresses exhibited by the formulations under study were probably a result of structural changes caused by shearing. The formulations consisted primarily of high-molecular weight components, organized in micelles with the water. Following exposure to shear stress, the dispersion could flow and the chains of the polymers could align along the direction of shear, releasing water, or water and propolis. As a result, subsequent shearing occurred more readily, and the apparent viscosity was decreased, favoring the flow, a fact that was reversed with the absence of shear stress. Furthermore, the low degree of thixotropy indicates that the restoration of the original configuration would require only a short time after removal of the shear stress (after the administration), therefore, enhancing retention therein.

Mucoadhesive polymers have been exploited for several decades by pharmaceutical scientists to formulate novel dosage forms for various routes of drug administration (buccal, oral, nasal, ocular, and vaginal). The research in this area continues to develop very quickly with more than a hundred new papers being published each year (for revision see [46]). The current efforts in this area are focused on the design of mucoadhesive polymers since the characteristic of most mucins is carrying a negative charge net due to the presence of carboxylic groups (sialic acid) and ester sulfates at the terminus of some sugar units. Additionally, the approximate p*Ka* of these acidic groups is 1.0–2.6 resulting in their complete ionization under physiological conditions [46], which is an interesting interface to the adherence of some polymers.

Regarding the mucoadhesive strengths presented here, it is possible to observe that the results obtained, that is, mucoadhesion for carbopol, poloxamer, sodium alginate, and chitosan polymers, are in accordance with other authors [10, 27, 47]. Although in some cases mucoadhesive strength was larger than 0.30 N, such as nonsteroidal anti-inflammatory gels evaluated by Barry [48], Perioli et al. [49] evaluated chitosan gels to vaginal application (0.5 and 2.0%) with 0.043 and 0.093 N of mucoadhesion. Moreover, Cid et al. [27] found values of 0.09–0.20 N for chitosan gels (1 and 3%). Considering that ChC- and ChP1% offered 0.33 and 0.37 N, respectively, the results obtained were very interesting and coherent since although ChC- and ChP1% possess 1.5% of chitosan, the effect was complemented with the presence of natrosol (cellulose derivate).

The results showed that carbopol was the most effective mucoadhesive polymer. Initially, it was expected that chitosan, due to its positive charges, from amino groups, could be the most interesting formulation to electrically adhere to vaginal mucous and epithelium (negatively charged); however, it was not successful. Moreover, concerning the long polymeric chain of carbopol, rich in carboxyl and hydroxyl groups, it was possible to foresee the numerous hydrogen bonds and Van der Waals forces that can be done between polymeric chain and charges of mucous and epithelium. This observation justifies the results obtained, in which the number of interactions of carbopol is higher than the interactions done by amino groups of chitosan; therefore, the effect observed is coherent. 

Results of physicochemical and *in vitro* biological characterization showed that all formulations were effective against all *Candida* strains. The rheological and mucoadhesive results suggest that CP1% and ChP1% were the most pseudoplastic formulations. CP1% and ChP1% were less viscous preparations while poloxamer was the most viscous gel. CP1% was the most mucoadhesive preparation, followed by both PP1% and ChP1%. All preparations possess low thixotropy. Taken together it is possible to suggest that all formulations can flow under shear stress, a fact that can be restored after the stopping of strength application (pseudoplastic behavior with little thixotropy). Considering this data, it is interesting to use a deformable packing to all preparations obtained. The viscosity and mucoadhesion can suggest that carbopol and poloxamer are interesting preparations to proceed biological efficacy tests.

Vaginally applied imidazoles are the first line treatment of vulvovaginal candidiasis. The need to develop a less inconvenient and more patient acceptable regimen led to the development of a treatment schedule from the original 3 weeks to 7 days protocols [50]. Currently, some topical preparations like clotrimazole 500 mg vaginal tablet, 2%, 6.5% tioconazole (Vagistat-1), and 2% miconazole ovule (Monistat1), among others, are currently available in USA. This class of compounds is fungicidal only at high concentrations and after prolonged incubation [51]. González et al. [52] evaluated voriconazole and fluconazole therapies by oral or topical route (0.5, 1, and 5 mg/kg once daily), where results showed that all therapy regimens administered topically for the treatment of vaginal infection significantly reduced the fungal load with respect to the negative control group (excipient). Considering topical treatments at analogous doses, voriconazole was as effective as fluconazole; nevertheless, neither drug administered orally nor topically was able to eradicate this microorganism from the vagina [52]. These results were corroborated by Stevens et al. [53] in topical treatments with clotrimazole and zeamantine treatments. Scott et al. [51] evaluated in systemically and topical murine models, a new class of antifungal agents, jasplakinolide, a structure derived from marine sponge. The topical administration of jasplakinolide 2% was very effective in reducing infection, with results showing 53% of negative cultures in this group, 47% with miconazole nitrate and 7% of the control (4 days after infection); however, the toxicity of this structure compromised its utilization. 

The analysis of the results presented here can suggest that some improvements in propolis formulations possibly can increase the response in animal treatments. Besides propolis concentration, maybe some changes in the thixotropy of the formulations or the presence of an oil phase can improve the delivery from propolis extracts of more lipophilic compounds from pharmaceutical systems. Actually, this was the way Bachhav and Patravale [54] improved fluconazole vaginal effects, offering completely eradication of the microorganism.

## Figures and Tables

**Table 1:** *Fingerprint *of chemical compounds of different propolis extracts evaluated (*n* = 3) (mg/g).

Samples	PEE		PMM		PSDE	
Standards	Mean	SD	Mean	SD	Mean	SD
Caffeic acid	0.345	0.011	ND	—	2.699	0.398
*p*-Coumaric acid	1.712	0.069	6.312	0.061	5.979	0.686
3,4-DCQ acid	0.488	0.006	1.138	0.014	5.842	0.132
3,5-DCQ acid	1.614	0.012	6.147	0.129	2.667	0.092
4,5-DCQ acid	3.442	0.023	13.308	0.203	7.430	0.045
Cinnamic acid	0.167	0.006	0.494	0.006	ND	—
Aromadendrin	0.808	0.024	2.628	0.024	ND	—
Artepillin C	6.621	0.306	6.593	0.348	51.384	6.036

ND: not detected.

**Table 2:** Minimum fungicidal concentration (MFC) of PEE, PWE, PMM, and PSDE for distinct *Candida* strains (*n* = 3).

Sample	MFC ± SD (mg/mL)		
	*C. albicans* SC5314	*C. parapsilosis *	*C. glabrata *
PEE	7.0 ± 0.0	7.0 ± 0.0	14.0 ± 0.0
PWE	24.5 ± 0.0	24.5 ± 0.0	24.5 ± 0.0
PMM	13.7 ± 0.0	27.5 ± 0.0	13.7 ± 0.0
PSDE	11.73 ± 0.0	11.73 ± 0.0	5.86 ± 0.0

**Table 3:** Yield exponent values to gel base and propolis gel (*n* = 3).

Samples	Gel base	Propolis gel
Polymers	Mean ± SD	Mean ± SD
Poloxamer 407 (Pluronic)	0.3845 ± 0.0098	0.4455 ± 0.0049
Sodium alginate	0.2425 ± 0.0041	0.2554 ± 0.0126
Carbopol 940	0.2750 ± 0.0060	0.2608 ± 0.0143
Chitosan	0.3326 ± 0.0193	0.3674 ± 0.0212
